# Spinal Cocainisation in Obstetrics

**Published:** 1903-03-21

**Authors:** 


### SPINAL COCAINISATION IN OBSTETRICS.

Dr. Cross, of Visalia, California, U.S.A., writes that, while his experience with spinal cocainisation in surgery has everything to commend its use, its employment in obstetrics is much more satisfactory, for it fills a more ideal position. It stops the pain of the patient without in any way diminishing the contractions of the uterus: the presenting part advances during the contractions of the uterus and does not recede when the pains stop, but retains its position, leaving the soft parts on the stretch. The labour is converted into a uterine labour entirely. The patient can assist the contractions of the uterus if requested to, but will not know when the contractions come if she is not told. Should any extended examination be required it can be made without pain. The soft parts are more easily manipulated, and lacerations of those structures are not so apt to result as under other conditions. If the forceps are to be used as much time can be occupied as is thought desirable. Dr. Cross himself has taken an hour and a half to two hours with the forceps in assisting a difficult labour. When the baby is born, the mother having been under the influence of spinal cocainisation, there is not so much cyanosis as usually occurs when chloroform has been used, and, moreover, hæmorrhage does not occur after delivery. The uterus continues to contract after the child is born, and the placenta is soon expelled with a strong contraction of the uterus following. Should laceration result, as much repair as is necessary can be done without causing any pain. The method of administration is as follows :—Four-tenths of a grain of sterile muriate of cocaine are placed in a Luer syringe, the puncture is made, the syringe attached, and enough spinal fluid drawn into the syringe to dissolve the cocaine ; when this has occurred the fluid is returned into the spinal canal. In from six to 20 minutes the patient is free from pain. A little nausea and sometimes vomiting, during which time the pulse may be weak, are the only bad effects Dr. Cross has noted ; when the nausea stops, the pulse comes to normal without any artificial stimulation. He does not wish to be understood as advocating the puncture in every case, but when any case requires an anæsthetic it is much better than chloroform or ether so far as his experience teaches. For using the forceps where the case is slow and the patient is becoming hysterical and exhausted the results are ideal. Where the perineum is tight and rupture seems certain the cocaine will often allow so much manipulation that laceration is avoided. In normal cases that are slow he has restricted its use to the time when the presenting part is well down near the perineum. He now finds many patients who know he uses the puncture request its use as soon as he is called, but if the labour is normal he does not use the puncture. In two years' experience with the puncture in both surgical and obstetrical work he has not oberved any bad results either immediate or remote. He regrets that his experience is not more extensive in its use, and hopes to hear from others who have had experience with it in obstetrical practice. The time the anæsthetic lasts is from one hour to four hours. Anyone who has seen a slow exhausting labour with much pain transformed into a not too rapid painless one can but be pleased with its use in such cases.

